# Influence of clinical factors, IL4 and IL6 genes polymorphisms in functional healing in late replantation

**DOI:** 10.1590/0103-6440202204683

**Published:** 2022-03-07

**Authors:** Liliane Roskamp, Cleber Machado Souza, Sergio Aparecido Ignácio, Camila Paiva Perin, Natanael Henrique Ribeiro Mattos, Isabela Roskamp Sunye, Letícia Capote Santos, Vania Portela Ditzel Westphalen, Carolina da Silveira Jacob, Flares Baratto-Filho

**Affiliations:** 1 Department of Dentistry, Universidade Tuiuti do Paraná - UTP, Curitiba, PR, Brazil.; 2 School of Life Sciences, Pontifícia Universidade Católica do Paraná - PUC/PR, Curitiba, Paraná, Brazil.; 3 Department of Dentistry, Universidade da Região de Joinville - Univille, Joinville, SC, Brazil.

**Keywords:** Genetic Polymorphism, Root resorption, Tooth avulsion, Tooth replantation

## Abstract

To investigate the genetic association in a sample of replanted teeth, it is necessary to observe the extreme phenotypes, such as, teeth that underwent functional healing and those extracted due to severe external root resorption. Thus, this study aimed to investigate the association of age of the patients, root development, storage media, and polymorphisms in the interleukin 4 (*IL4*) and interleukin 6 (*IL6*) genes with teeth that presented extreme outcomes, as functional healing or extraction, in a group whose replantation techniques did not follow the International Association of Dental Traumatology (IADT) 2012 guidelines. Forty-three avulsed and replanted teeth that did not follow IADT 2012 guidelines and underwent functional healing or were extracted were included. Periapical radiographs employed for this study were taken soon after tooth replantation and after 1 year. For genotypic *IL4* and *IL6* genes analysis, DNA of oral mucosa cells was extracted. Real-time- PCR performed for genotyping polymorphisms in *IL4* and *IL6* genes. Clinical and genetic variables were analyzed by the Chi-square test and the “Z” test. P values < .05 were considered significant. The results showed that functional healing and extraction were associated with storage media and with the rs2243268 of *IL- 4* gene polymorphisms. As conclusion, the C rs2243268 allele of *IL4* gene may have a positive relationship with functional healing teeth that were replanted not following the 2012 IADT guidelines. Keeping the tooth dry is associated to a fast loss of avulsed and replanted teeth after 1-year follow-up.

## Resumo

Para investigar a influência genética em uma amostra de dentes reimplantados, é necessário observar os fenótipos extremos, como os dentes que sofreram cura funcional e os extraídos devido à reabsorção radicular externa severa. Assim, este estudo teve como objetivo investigar a associação da idade dos pacientes, desenvolvimento radicular dos dentes, assim como o meio de transporte até o reimplante e polimorfismos nos genes da interleucina 4 (IL4) e da interleucina 6 (IL6) com dentes que apresentaram cura funcional ou extração, em um grupo cujas técnicas de reimplante não seguiu a International Association of Dental Traumatology (IADT) 2012. Foram incluídos 43 dentes avulsionados e reimplantados que não seguiram as diretrizes do IADT, e tiveram cura funcional ou foram extraídos. As radiografias periapicais utilizadas para este estudo foram feitas logo após o reimplante dentário e após 1 ano. Para a análise genotípica dos genes *IL4 e IL6*, foi extraído DNA de células da mucosa oral. PCR em tempo real realizou a análise dos polimorfismos dos genes. As variáveis ​​clínicas e genéticas foram analisadas pelos testes Qui-quadrado e “Z”. Valores de p <0,05 foram considerados significativos. Os resultados mostraram que a cura ou perda dos dentes está associada ao meio de armazenamento e polimorfismos no gene rs2243268 da *IL-4.* Como conclusão, o alelo C rs2243268 do gene *IL4* pode ter uma relação positiva com a cura functional do dente reimplantado. Manter o dente seco está associado a uma perda rápida de dentes avulsionados e reimplantados que não seguiram o IADT 2012.

## Introduction

According to the literature, about 15% of dental trauma result in avulsion, and the higher incidence occurs in children between 7 and 15 years-old ^1^. Most rescuers do not know how to handle the avulsed tooth. Sometimes, even the general dentist does not correctly manage the replantation technique and, if these teeth are not properly treated, irreversible consequences as root resorption can occur, ending in tooth extraction ^2^ ^,^ ^3^. Therefore, these injuries have become a public health problem ^1^.

Resorption happens mainly when a total or even a partial loss of the periodontal ligament occurs, especially when the optimum condition for successful replantation is not present ^3^. Avulsion of the tooth trigger an inflammatory reaction, and its intensity, aggravated by the penetration and proliferation of bacteria in the local environment will determine the consequences on the root surface. Thus, the host immune-inflammatory response, and the balance of anti and pro-inflammatory cytokines may have a fundamental importance in the maintenance of the tissue health ^4^ ^,^ ^5^ ^,^ ^6^ ^,^ ^7^. These cytokines are coded by genes, and Single Nucleotide Polymorphisms (SNPs) are the most common forms of variation in deoxyribonucleic acid (DNA) in the human genome ^8^. They can compromise the amount or function of the expressed protein and influence the susceptibility or protection of external root resorption ^9^.

Teeth whose replantation follow the International Association of Dental Traumatology (IADT) guidelines, are expected to have the best outcomes ^10^. Since it is the first report of a genetic association of replanted teeth that didn´t followed the 2012 IADT guidelines ^10^ and even though some teeth remained healthy, it aims to raise some questions upon the importance of gene polymorphisms.

Therefore, this study aims to investigate the association of age of the patients, root development, storage media and polymorphisms in *IL4* and *IL6* genes with the replantation outcomes of teeth not following the IADT 2012 guidelines in 1-year of clinical and radiographic follow-up.

## Materials and methods

### Population Study

**Figure 1:**
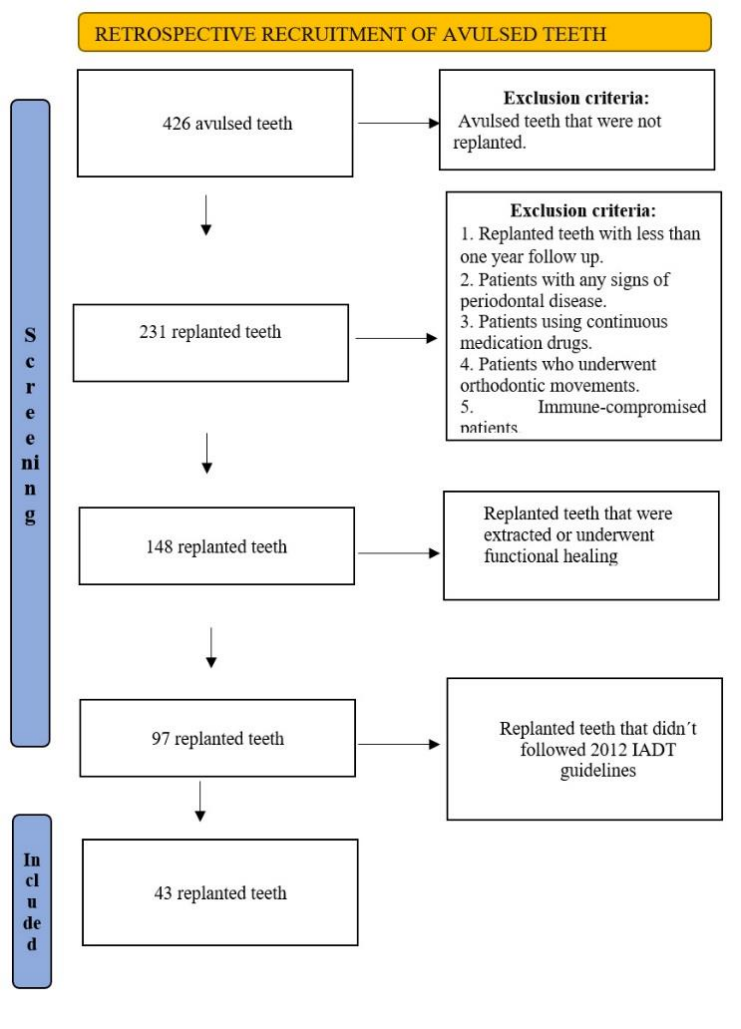
Chart flow diagram

Two hundred and thirty-one avulsed and replanted teeth had their root canal treated in a Dental Trauma Clinic were selected. Of them, a sample of 43 avulsed teeth whose replantation did not follow IADT 2012 guidelines composed this research (Figure 1). All teeth were replanted in the same day of the accident. Even though all of them were improperly stored for more than 1 hour of extra alveolar time, because of a lack of knowledge of the general dentists, they were just replanted as they arrived at the office. They didn´t have their root surface cleaned, neither with a gauze nor with any chemical substance, and no root canal dressing was performed before replantation, as the IADT 2012 guidelines suggested for late replantation after avulsion. A flexible splint at the buccal surface of at least 4 teeth was placed, and antibiotic, anti-inflammatory, a painkiller, and a tetanus buster, were prescribed. All patients were then referred to a Trauma Dental Clinic.

All teeth were treated with the same endodontic technique in the same Dental Clinic. In the first visit, from 7 to 21 days after the emergency care, all teeth were cleaned and shaped, and a calcium hydroxide dressing was placed inside the root canal. The follow up was every three months. After 1 year follow-up, the replanted teeth were divided into 2 groups: i) control group: teeth that underwent functional healing (teeth without signs and/or symptoms of external root resorption) and ii) case group: extracted teeth due to severe external resorption. Both groups were considered extreme outcomes. Objectives and procedures of this study were explained to all patients and an Informed Consent was signed. This work was approved by the local Research Ethics Committee, under number CEP 02320084000-10 and it followed the checklist of the Strengthening the Genetics Association Study Report (STREGA) statement ^11^.

### Inclusion Criteria

Patients of both sexes, whose avulsed teeth did not follow IADT 2012 guidelines at the first care replantation management. Patients whose teeth underwent a functional healing or those who have lost their replanted teeth due to external root resorption in a 1-year follow-up. Patients with good oral hygiene, without any signs of periodontal disease or any other soft or hard tissue diseases in the mouth. Patients who did not use continuous medication drugs. Patients who did not undergo orthodontic movements. Patients that were not immune compromised by autoimmune diseases, chemotherapy, infectious diseases, parasitic or immunodeficiency.

### Radiographic and Clinical Data

The periapical radiographs of the replanted teeth were performed with the aid of radiographic positioners JON (São Paulo-SP, Brazil), at their first appointment at the Trauma Dental Clinic and after 1 year. Two calibrated PhD endodontists evaluated the teeth independently, clinical and radiographically, with a very high concordance level: a very high intraexaminer agreement (Kappa scores: 0.98% and 0.95%) and a very good interexaminer agreement (0.95%). Radiographic signs such as: presence or absence of root resorption, complete presence, total or partial interruption of lamina dura, ankylosis, mobility, responses to horizontal and vertical percussion, presence or absence of spontaneous pain or pain on palpation were carefully evaluated. Only the teeth that did not present any signs of external root resorption/ankylosis, that is, that underwent a functional healing and teeth that were extracted due to external root resorption were selected to compose the analysis. All radiographs received a number, to assure a blind evaluation.

The age was divided in three categories: permanent teeth from children up to 12 years; from 13 to 18 years and older than 19 years. Teeth with closed or open apices composed the root development group. Teeth kept dry, in saline or in an unknown medium except milk, composed the storage media group. The Hank´s Balanced Salt Solution (HBSS) is not available in our region.

### Collection and DNA Purification

The DNA collection, purification and the analysis of the polymorphisms were performed in a laboratory of the same institution where the patients received the endodontic treatment and follow up. The selected patients rinsed for 1 minute with a glucose solution 3%. After rinsing, a sterile wooden spatula was used to scrape the buccal mucosa ^12^. The tip of the spatula was then agitated in the rinsed solution. Buccal epithelial cells were pelleted by centrifugation at 2000 rpm for 10 minutes. The supernatant was discarded, and the pellet suspended in 1300 µL of extraction buffer [10 mM Tris-HCl (pH 7.8), 5 mM EDTA, 0.5% SDS (Biotec, Sao Paulo, SP, Brazil)]. Then 10 µL proteinase K (20 mg/mL) (Invitrogen, Waltham, MA, USA) was added to the solution and left overnight at 65°C. DNA was purified by the addition of 10 M ammonium acetate, precipitated with isopropanol, ethanol 70% and suspended in 50 µL of 10 mM Tris (pH 7.6) and 1 mM EDTA ^12^. After this extraction process, the DNA was kept at -20°C. The collect data received a number, as well as each radiograph, to assure a blind evaluation.

### Analysis of the Polymorphisms in the IL4 and IL6 Genes

SNPs in high linkage disequilibrium (LD) allows the researcher to genotype all polymorphisms in "target" SNPs (tag SNP), structure that captures all gene information in terms of variability ^8^. *IL4* and *IL6* tag SNPs were selected, employing some criteria, such as population minimum allele frequency (MAF) of 5% and 80% cutoff (r2<0.8) to define the LD, according to the information available on the site International HapMap Project, phase III/Rel#2 (http//www.hapmap.org, 2014) ^13^. Two tag SNPs, capturing all information of *IL4* gene: reference SNP (rs) rs2227284 and 2243268, and 8 tag SNPs for *IL6* were selected: rs1524107, rs2069835, rs2069837, rs2069838, rs2069840, rs2069842, rs2069843 and rs2069845. They were genotyped by Real-Time Polymerase Chain Reaction (RT-PCR), using the apparatus of Applied Biosystems 7500 (7500 Real-Time PCR System) with the TaqMan™ Genotyping Master Mix (Applied Biosystems, Foster City, CA, USA). The genotyping success rate was ≥ 95% for all markers tested. After that each SNP was assessed for genotypic modes of transmission (additive, dominant and recessive models).

### Statistical Analysis

The following variables were studied: demographic and clinical variables (age, root development, storage media) *IL4* (SNP rs2227284 and 2243268) and *IL6* (SNP rs1524107, rs2069835, rs2069838, rs2069840, rs2069842, rs2069843, and rs2069845) polymorphisms. To estimate the Hardy-Weinberg equilibrium and evaluate the LD, 4.2 Haploview software was employed. Statistical analyses were performed by the 25.0 SPSS version (IBM Corp., Armonk, NY, USA). The variables were analyzed by Chi-square test, the “Z” test and Fisher's exact test. P values < .05 were considered significant. Values of the “Likelihood Ratio”, when the expected frequency was smaller than 1.0, were chosen. For univariate genetic analyses, the correction for multiple tests was performed. This calculus was automatically made by the 25 SPSS version. (Chi-square Test and “Z” Test of differences between two proportions with Bonferroni correction). Missing data were included as missing values.

## Results

### Clinical Analysis

In the non-IADT group, 24 (55.8%) teeth underwent functional healing, and 19 (44.2%) were extracted during the first year of replantation.

There was a significant difference for storage media (teeth kept dry, in saline solution or in an unknown medium) between and within the functional healing and extraction (P < .05). There was no significant difference for age or root development (P > .05) (Table 1).

### Genetic Analysis

The genotype frequencies of the tested tag SNPs were in Hardy-Weinberg equilibrium in the control group. The analysis of LD between markers confirmed independence among them (r2<0.8). There was a significant difference (P < .05) for *IL*4 rs 2243268 between the groups only in the dominant model (Table 2). No significant difference was observed for *IL4* rs2227284 or any of the tag SNPs of *IL6* gene in the dominant, recessive, or addictive models analyzed (P>.05). There were 2 missing values in rs2069835, and one in rs2069845. (Tables 2 and 3).

**Table 1:** Relationship between functional healing and tooth extraction x “age range, root development and storage media” of replanted teeth in 1 year follow-up.

n=43	Age range (*p* > .05)			Root development (*p* > .05)		Storage media (*p* < .05)			Total
	<_ 12 years	13-18 years	>_19 years	closed apex	open apex	dry	saline solution	unknown	
Functional healing	16_a_	5_a_	3_a_	21_a_	3_a_	14_a_	8_b_	2_a, b_	24
	66,7%	20,8%	12,5%	87,5%	12,5%	58,3%	33,3%	8,3%	100,0%
Extraction	9_a_	6_a_	4_a_	17_a_	2_a_	18_a_	1_b_	0_a, b_	19
	47,4%	31,6%	21,1%	89,5%	10,5%	94,7%	5,3%	0,0%	100,0%
Total	25	11	7	38	5	32	9	2	43
	100,0%	100,0%	100,0%	100,0%	100,0%	100,0%	100,0%	100,0%	100,0%

Each different subscript letter denotes a subset of age range, root development and storage media categories whose column proportions differ significantly from each other at the .05 level. (Chi-square Test and “Z” Test of differences between two proportions with Bonferroni correction). Age range (*P* = .44); root development (*P* = .84); storage media (*P* = .01)

**Table 2:** Genotype analysis of IL4 gene tag SNPs in dominant, recessive, and addictive models for the most uncommon alleles.

Tag SNPa	Variation	Genetic Model	Groups	Genotype (%)			Univariate	
	[1/2]^b^			Homozygous 1	Heterozygous	Homozygous 2	*p-* * Value	OR (CI 95%)
rs2227284 [G/T]		Additive	Functional healing	10_a_ (41.7)	6_a_ (25.0)	8_a_ (33.3)	0.143	
			Extraction	8_a_ (42.1)	9_a_ (47.4)	2_a_ (10.5)		
		Recessive	Functional healing	8_a_ (33.3)	16_a_ (66.7)		0.145	4.2 (0.8 -23.1)
			Extraction	2_a_ (10.5)	17_a_ (89.5)			
		Dominant	Functional healing	10_a_ (41.7)	14_a_ (58.3)		0.977	1.0 (0.3- 3.3)
			Extraction	8_a_ (42.1)	11_a_ (57.9)			
rs2243268 [A/C]		Additive	Functional healing	12_a_ (50.0)	9_a_ (37.5%)	3_a_ (12.5)	0.094	
			Extraction	15_a_ (78.9)	4_a_ (21.1%)	0_a_ (0.0)		
		Recessive	Functional healing	3_a_ (12.5)	21_a_ (87.5)		0.110	0.9 (0.8- 1.0)
			Extraction	0 (0.0)	19 (100.0)			
		Dominant	Functional healing	12_a_ (50.0)	12_a_ (50.0)		0.051	0.267 (0.1 - 1.0)
			Extraction	15_a_ (78.9)	4_a_ (21.1)			

a identification of SNP based in NCBI dbSNP; b the first letter indicates the common allele and the second is the most uncommon; * Pearson Chi-Square Test. Each different subscript letter denotes a subset category whose column proportions differ significantly from each other at the .05 level. (Chi-square Test and “Z” Test of differences between two proportions with Bonferroni correction).

**Table 3:** Genotype analysis of *IL6* gene tag SNPs dominant, recessive, and additive models for the most uncommon alleles.

Tag SNPa	Variation	Genetic Model	Groups	Genotype (%)			1.	Univariate
	[1/2]b			Homozygous1	Heterozygous	Homozigoto 2	*p*-Value*	OR (CI 95%)
rs1524107	[C/T]	Additive	Functional healing	20_a_ (83.3)	4_a_ (16.7)	0_a_ (0.0)	0.250	2.3 (0.5-9.8)
			Extraction	13_a_ (68.4)	6_a_ (31.6)	0_a_ (0.0)		
rs2069835	[T/C]	Additive	Functional healing	18_a_ (75.0)	4_a_ (16.7)	0_a_ (0.0)	0.151	
			Extraction	16_a_ (84.2)	0_a_ (0.0)	0_a_ (0.0)		
rs2069838	[C/T]	Additive	Functional healing	24_a_ (100)	0_a_ (0.0)	0_a_ (0.0)		
			Extraction	19_a_ (100.0)	0_a_ (0.0)	0_a_ (0.0)		
		Recessive	Functional healing	0_a_ (0.0)	24_a_ (100)			
			Extraction	0_a_ (0.0)	19_a_(100.0)			
		Dominant	Functional healing	24_a_ (100)	0_a_ (0.0)			
			Extraction	19_a_ (100.0)	0_a_ (0.0)			
rs2069840	[C/G]	Additive	Functional healing	9_a_ (37.5)	15_a_ (62.5)	0_a_ (0.0)	0.504	
			Extraction	6_a_ (31.6)	12_a_ (63.2)	1_a_ (5.3)		
		Recessive	Functional healing	0_a_ (0.0)	24_a_ (100)		0.255	1.1 (1.0- 1.2)
			Extraction	1_a_ (5.3)	18_a_ (94.7)			
		Dominant	Functional healing	9_a_ (37.5)	15_a_ (62.5)		0.686	1.3(0.4- 4.6)
			Extraction	6_a_ (31.6)	13_a_ (68.4)			
rs2069842	[G/A]	Additive	Functional healing	23_a_ (95.8)	1_a_ (4.2)	0_a_ (0.0)	0.368	1.0(0.9-1.0)
			Extraction	19_a_ (100.0)	0_a_ (0.0)	0_a_ (0.0)		
rs2069843	[G/A]	Additive	Functional healing	19_a_ (79.2)	4_a_ (16.7)	1_a_ (4.2)	0.320	
			Extraction	18_a_ (94.7)	1_a_ (5.3)	0_a_ (0.0)		
		Recessive	Functional healing	1_a_ (4.2)	23_a_ (95.8)		0.368	1.0 (0.9- 1.0)
			Extraction	0_a_ (0.0)	19_a_ (100.0)			
		Dominant	Functional healing	19_a_ (79.2)	5_a_ (20.8)		0.143	0.2 (0.0 - 2.0)
			Extraction	18_a_ (94.7)	1_a_ (5.3)			
rs2069845	[A/G]	Additive	Functional healing	12_a_ (50.0)	7_a_ (29.2)	5_a_ (20.8)	0.304	
			Extraction	9_a_ (47.4)	8_a_ (42.1)	1_a_ (5.3)		
		Recessive	Functional healing	5_a_ (20.8)	17_a_ (70.8)		0.161	
			Extraction	1_a_ (5.3)	17_a_ (89.5)			
		Dominant	Functional healing	12_a_ (50.0)	12_a_ (50.0)			
			Extraction	9_a_ (47.4)	9_a_ (47.4)			

a  identification of SNP based in NCBI dbSNP; ^b^ the first letter indicates the common allele and the second is the most uncommon; * Pearson Chi-Square Test. Each different subscript letter denotes a subset of categories whose column proportions differ significantly from each other at the .05 level. (Chi-square Test and “Z” Test of differences between two proportions with Bonferroni correction). There were 2 missing values in rs2069835. There was one missing value in rs2069845.

## Discussion

This is the first study that employed an approach analyzing demographic, clinical aspects, and performed a complete physical mapping of *IL4* and *IL6* genes in a sample of 43 teeth whose replantation didn´t follow the 2012 IADT guidelines and presented a functional healing or were extracted due to a fast and intense external root resorption.

The IADT does not guarantee dental replantation favorable results, however, it emphasizes that, following them, the maximization of success can occur. However, the dentist takes the final treatment judgment ^14^. The lack of knowledge of general population on how to handle an avulsed tooth resulted in this sample. Patients who composed this study arrived at the Trauma Dental Clinic to have their root canal treated with their teeth already replanted, some of them 21 days before. All teeth were kept in unsuitable media, as saline solution, or were kept dry for more than one hour, and their root surfaces were not cleaned before replantation. It means that the late replantation technique suggested by 2012 IADT guidelines for teeth kept in an unsuitable medium for more than 1 hour was not followed ^12^. Dead cells and fibers of periodontal ligament were attached to avulsed teeth during emergency replantation procedures. The unviable periodontal ligament was carried to the socket and consequently an immuno-inflammatory reaction next to the root surface took place, harming the surrounding tissue ^6^.

The functional and aesthetics significance of a replanted tooth, associated with young age of patients, encourage the search for more effective solutions, regarding the best treatment in late replantation, knowledge, and prevention of likely causes of external root resorption. Even though IADT suggests a technique for late replantation, the ideal method is neither yet established nor is this information worldwide spread. Continuous educational campaigns on replantation should be a goal of health care entities and governments.

Patients in this study were mostly younger than 19 years old. According to the literature, younger patients suffer more dental avulsion than adults ^2^ . However, no significant difference was found for the age between the groups in accordance with Petrovic et al. study ^15^. Even though 21 of 36 replanted teeth of patients younger than 19 years old established a functional healing, no significant difference was found, differently as the results by Bastos et al. ^16^, in which patients older than 16 years had significant less chance of developing root resorption. In our study, age was divided in three ranges based in the following rational: i) permanent teeth of children up to 12 years (the child is still changing its deciduous to permanent dentition); ii) from 13 to 18 years (even though the face is developing, there is no more tooth replacement and the patient is in his best moment of immunological response), and iii) after 19 years (the bone structure is already defined, and only the bone remodeling will continue through life). We suggest that the separation of the patients in those age ranges may contribute to point out the real importance of age in patient’s development, consequently, in root resorption.

In this study, it was not found an association for root development with the outcomes as also suggested by others ^17^, differently from the results with immature incisors, which exhibited more complications compared with mature teeth ^15^.

It is well known that non-favorable media, such as keeping the tooth dry, in saline or saliva present bad prognosis, with high rate of root resorption or extraction ^18^. Results of this study agree with other authors ^18^ ^,^ ^19^. Authors suggest the importance of the status of periodontal ligament on healing of replanted teeth ^4^ ^,^ ^6^ ^,^ ^17^. Indeed, these results also demonstrate that, to achieve a better performance in late replantation, the development of novel solutions to maintain or re-establish the viability of periodontal ligament must be focus for new research. Besides, educational campaigns are of great importance to teach the population never keep the avulsed tooth dry until replantation.

Stability or progression of periapical lesions are determined by the balance between pro and anti-inflammatory mediators, in modulation of receptor activator of NFkB ligand (RANKL), receptor activator of NFkB (RANK), and its receptor antagonist, osteoprotegerin (OPG) ^6^. IL-4 is one of the anti-inflammatory cytokines that initiate, maintain and increase T helper 2 lymphocyte responses (Th2) ^6^. It acts as a protective mediator of root resorption because of its ability to increase OPG levels and to inhibit RANK. It suppresses pro-inflammatory T helper 1 (Th1) cytokines, decreasing macrophage/clasts action ^6^. The regulatory T cells (Tregs) and Th2 cytokines such as IL-4 are associated with the attenuation of periodontal disease ^21^ and inactivity of periapical lesions ^22^ and functional healing in tooth replantation ^4^ ^,^ ^6^.

Polymorphisms of *IL4* gene have been studied to assess their role in the destruction of hard tissue. ^18^ ^,^ ^19^. Authors, in a larger sample of replanted teeth, report that *IL4* gene polymorphisms are not associated with external root resorption, when teeth are replanted according to IADT guidelines ^18^ ^,^ ^19^. The difference between this research is the correct technique employed during replantation procedures in the above mentioned and the very incorrect manipulation in the present study, where it was observed a significant difference in the rs2243268 and “functional healing and extraction”, suggesting that the C allele may have a positive relationship with functional healing. Besides, as IL-4 is an anti-inflammatory cytokine, modulating the inflammatory response may contribute to the maintenance of a healthy tooth in its socket. Maybe the strongness for the anti-inflammatory cytokine IL-4 protects the teeth to develop root resorption in patients presenting the polymorphism; however, this result must be seen with caution.

IL-6 is a pleiotropic cytokine, acting as a pro-inflammatory cytokine, which activates clasts, promoting hard tissue resorption in the presence of infections ^23^. It was suggested as a severe inflammatory cytokine of chronic apical lesions ^23^.

The polymorphisms of the *IL6* gene affect circulating IL-6 levels. The C allele revealed change in the gene transcription in response to stimuli such as lipopolysaccharide (LPS) ^24^. There was a significant association between allele G or GG genotype for the polymorphism -174 (G/C), also known as rs1800795, of the *IL6* gene and its presence in acute dental abscesses ^24^. In addition, the rs2069843 polymorphism of*IL6*may influence the outcome of avulsed and replanted teeth in the first-year post-trauma ^9^. In the present study, no significant association between *IL6* gene and root resorption outcomes was observed, even though a study with patients orthodontically treated, resulted with a significant association between rs2069843 and external apical root resorption ^25^.

When this research began, the IADT 2012 was the last guidelines published ^10^. It suggested that the root should be cleaned to remove the PDL, and the cleaning, shaping and calcium hydroxide dressing placed before late replantation ^10^. In the last 2020 guidelines, it is acceptable to replant the tooth without handling the root surface, and cleaning, shaping and root canal dressing placed after the splinting, in the same appointment of the emergency care or in the next one ^14^. In this manuscript, we included teeth that didn´t have their PDL removed in the emergency care and the root canal was performed in the next appointment, as it is possible as stated by the 2020 IADT guidelines ^14^. However, there are some other differences, as splinting time ^10^ ^,^ ^14^. For this reason, it is stated that no guidelines were really followed.

This research emphasis the importance of avoiding the dry storage of the avulsed tooth until replantation, since there was a significant difference when it was wrapped in a paper sheet or handkerchief. The storage of avulsed teeth in milk is associated with enhanced tooth survival ^18^, and therefore, it is one of the best easily available storage media, when the tooth is not immediately replaced in its socket.

This study has some limitations. The number of replanted teeth that underwent a wrong management of replantation technique is small. This seems to be good, since an intense educational campaign was performed in the city some years ago. However, it is unsatisfactory for a genetic study. The development of root resorption may have a strong interference of the handling and treatment of the replanted tooth along with a genetic influence ^6^ ^,^ ^9^ ^,^ ^10^ ^,^ ^14^. Therefore, it is difficult to establish a direct relationship between phenotype and genotype. For this reason, this study selected a sample completely unfavorable to the maintenance of healthy teeth, demonstrating a strong control group, that means, teeth that underwent functional healing, even though their management did not follow the IADT 2012 guidelines.

Why these teeth remained without resorption after 1 year, even though their cells and periodontal ligament were impaired during the replantation procedures? Maybe the key factor lies in the favorable immuno-inflammatory reaction of the host.

## Conclusion

The C rs2243268 allele of *IL4* gene may have a positive relationship with functional healing in teeth that were replanted not following the 2012 IADT guidelines. Keeping the tooth dry is associated to a fast loss of avulsed and replanted teeth in 1-year follow-up.
